# Less Illusion of a Just World in People with Formally Diagnosed Autism and Higher Autistic Traits

**DOI:** 10.1007/s10803-020-04831-7

**Published:** 2020-12-23

**Authors:** Alex Bertrams

**Affiliations:** Educational Psychology Lab, Faculty of Human Sciences, Institute of Educational Science, University of Bern, Fabrikstrasse 8, 3012 Bern, Switzerland

**Keywords:** Autism, Autistic traits, Belief in a just world, Locus of control, Self-deception

## Abstract

People differ in how strongly they believe that, in general, one gets what (s)he deserves (i.e., individual differences in the *general belief in a just world*). In this study (*N* = 588; *n* = 60 with a formal autism diagnosis), whether or not autistic people and those with high autistic traits have a relatively low general belief in a just world is examined. The results revealed the expected relationship between autism/higher autistic traits and a lower general belief in a just world. In a subsample (*n* = 388), *personal* belief in a just world, external locus of control, and self-deception mediated this relationship. These findings are discussed in terms of autistic strengths (less biased information processing) and problems (lowered well-being).

## Introduction

Orson Welles is reported to have once said, “Nobody gets justice. People only get good luck or bad luck.” While we do not know whether Welles would have been diagnosed with autism, it has been argued that he might have been on the autism spectrum, given some biographical notes about his traits and behaviors (Ledgin 2002). Welles’s autism-like personality and his views about justice might not have coincided purely by chance; there is possibly an underlying general regularity. In this sense, this work aimed to research whether Welles’s quote reflects a view that is common amongst autistic people and people high in autistic traits.

### General Belief in a Just World

From a scientific perspective, there is no reason to assume that the world is generally just. In other words, there is no real evidence for a natural law within the universe that, in general, people get what they deserve and deserve what they get (the core idea of a just world; Lerner and Miller 1978; Lipkus 1991). In addition to the subjectivity of the cultural construct “deserving,” many examples speak against justice as a principle of nature (e.g., the cruel deaths of little children reported in the news or history books). Still, many people stick to the idea of a just world, which has been extensively examined by social psychologists under the term *(general) belief in a just world* (Furnham 2003; Lerner 1980; Rubin and Peplau 1975; Wolfradt and Dalbert 2003).

One important reason for the general just world belief is that it can buffer the stress connected with the unpredictability and threats of life (Furnham 2003; Lerner 1980). When believing the world is just, the future appears to be more controllable if acting according to accepted standards. For instance, for keeping the Ten Commandments (one possible standard people take), one could expect deserving to remain spared from the strokes of fate. In this view, the general belief in a just world is a positive, self-serving illusion, and individual differences in this belief have been found to be related to well-being (Dalbert 1998; Yu et al. 2018). However, the downside of believing in a just world is that it can also cause the rejection and blaming of (innocent) victims (Hafer 2002; Lerner and Simmons 1966; van den Bos and Maas 2009). Inherent to the logic of the stress-buffering effect, victimhood is considered to result from the violation of an important standard, meaning victims deserve their fate to some extent. In summation, believing that the world is generally just is a fallacy that can have both desirable and undesirable implications.

### Autism and Autistic Traits

Autism is diagnosed based on difficulties in social communication and interaction, as well as the occurrence of restricted and repetitive behaviors (American Psychiatric Association 2013). Much research has also been focused on autistic traits (i.e., particularities in communication, social skills, imagination, attention to detail, and attention switching; Baron-Cohen et al. 2001) as individual difference variables in non-clinical populations (e.g., Albantakis et al. 2020; Bertrams and Schlegel 2020; Goris et al. 2020). Some authors have made arguments regarding similarities in the behaviors and experiences of formally diagnosed autistic people and people high in autistic traits without a formal diagnosis (e.g., Poljac et al. 2012). However, the groups should not be viewed as equivalent, as not all people high in autistic traits qualify for the formal diagnosis of autism (Happé et al. 2016; see also Mottron and Bzdok 2020 for profound criticism of the study of autistic traits in contemporary autism research). Therefore, in autism research, it makes sense to look at both groups in considering whether some hypothesized relationship can be determined (e.g., Brosnan et al. 2016).

### Autism/High Autistic Traits and Lowered General Belief in a Just World

This study investigated the hypothesis that formally diagnosed autistic people, as well as people relatively high in autistic traits but without a formal autism diagnosis, possess a lower general just-world belief than non-autistic people or people who are low in autistic traits, respectively. This supposed difference in world views could mean that people with autism and high autistic traits tend to be more realistic and, somewhat ironically, sometimes view victims fairer than their non-autistic counterparts. Indeed, less-biased information processing has been argued and demonstrated to be a typical characteristic of autistic people and those high in autistic traits (Bayesian explanatory approach: Karvelis et al. 2018; Pellicano and Burr 2012; Dual Process Theory of autism: Brosnan et al. 2016; Lewton et al. 2019). In this sense, this study may relate to reflections on autistic strengths (e.g., Mottron 2011). On the other hand, it could mean that autistic people and those high in autistic traits do not make use of a strategy for self-regulating their negative affect, which could be one reason why autism and high autistic traits are associated with reduced well-being (Lord et al. 2020; Stimpson et al. 2020). (In this regard, notably, that low well-being is more typical when the behavioral manifestation of autism is relatively subtle, while more severe behavioral manifestations of autism are usually related to higher well-being within the autism spectrum; Kapp 2018.) Three potential reasons for the assumed negative relationship between autism/autistic traits and the general just world belief were considered in this work.

#### Lower Personal Belief in a Just World

First, it may be that being autistic/high in autistic traits is also related to a lower *personal* belief in a just world. While the general belief in a just world refers to the extent to which individuals believe that the world is generally a just place, the personal belief in a just world reflects the extent to which they believe that they are treated fairly (Dalbert 1999). The personal and general beliefs in a just world are two distinct variables that are moderately related (Dalbert 1999; Nudelman et al. 2016).

Autistic people and people high in autistic traits may find themselves personally confronted with treatment and events that are not always fair and deserved. The physical environment and many social norms are generally not adapted to the needs of the autistic minority. Therefore, even with high motivation and potential to perform, autistic people struggle with sensory, communication, and interaction problems in typical working environments and often remaining unemployed (Lorenz et al. 2016). At the same time, they may see how non-autistic people get along with much less effort. Similar inequality may be given in other domains of life, such as romantic relationships (Strunz et al. 2016). Moreover, autistic people are particularly at risk of school bullying victimization (Maïano et al. 2016), leaving them likely confused as to why they deserve such treatment. Insofar as these problems have to do with autistic traits (e.g., particularities in communication; Baron-Cohen et al. 2001), non-diagnosed people with high autistic traits should also be affected. If adverse experiences such as these cause a lower personal belief in a just world, it could lead affected persons to hold less of a belief that the world is just in general.

#### Higher External Locus of Control

Second, the same reasons why autistic people and people high in autistic traits are assumed to have a lowered personal belief in a just world may contribute to a higher external (or lower internal) locus of control. Having an external locus of control means that the environment, including external rewards and punishments, is seen as personally uncontrollable. On the opposing side of this continuum, having an internal locus of control is the belief that the environment is responsive to one's own relatively permanent characteristics and that one can determine one’s own rewards and punishments, rather than being dependent on external forces (Rubin and Peplau 1975; Wang and Lv 2017).

Autistic people can face difficulties in achieving some of their goals/dreams (e.g., getting or keeping a job, finding a partner) due to incompatibilities between themselves and their social environments during communication and interaction (Lorenz et al. 2016; Strunz et al. 2016). Such failures may contribute to the development of an external locus of control (see Rotter 1966). Receiving unwanted, harmful treatment from the social environment (e.g., Maïano et al. 2016) should also increase an external relative to an internal locus of control. In addition, Hillier et al. (2020) argued that autistic people perceive their control options as relatively low due to negative stigmatization factors; namely, they are more likely to receive negative signals regarding their right to make choices and control situations around them. Even though not all autistic people and people high in autistic traits may have such negative experiences, one can expect them to exhibit, on average, an elevated external locus of control.

An external locus of control has consistently been substantially related to a lower general belief in a just world (Rubin and Peplau 1975). One reason for this relationship may be that the stress-buffering effect of the just-world belief would require the subjective view that personally acting according to accepted standards is generally effective.

#### Lower Self-deception

The relatively reduced tendency to deceive oneself may be a third reason for a lower general belief in a just world. Several studies indicate that autistic people have an impaired ability for deceptive behavior (see Abe 2011). The same could be true for people high in autistic traits (Jameel et al. 2014). Recent research has called into question whether autistic people are less deceptive by showing that autistic and non-autistic people did not differ in their scores on the Marlowe-Crowne social desirability scale (Gernsbacher et al. 2020). However, in the previous autism-related research, scholars have not distinguished between the conscious deception of others (i.e., impression management) and the rather non-conscious self-deception, which may have caused some inconsistency. The Marlowe–Crowne scale does not distinguish between the two factors and more strongly represents the conscious impression management dimension (Paulhus 1991). Given that autism and autistic traits are related to a thinking style that is based on facts and rational rather than intuitive thought (Brosnan et al. 2016; Lewton et al. 2019), it can be expected that autism and higher autistic traits are related to lower self-deception.

Self-deception can be understood as information-processing biases that give priority to welcome over unwelcome information, involving selective searching for and attention to information, misremembering, misinterpretation, and rationalization (von Hippel and Trivers 2011). In this way, self-deception has a beneficial function for the self and can foster self-confidence and optimism. Believing that the world is generally just may be one piece of self-deception for the sake of protecting one's subjective well-being. In case that autistic people and people high in autistic traits are less susceptible to self-deception, they should also be less prone to belief in a just world. In other words, it is assumed that self-deception mediates the suspected negative relationship between autism/autistic traits and the general belief in a just world.

### The Present Research

Based on this rationale, in this study, the following hypotheses were examined:

1. On average, formally diagnosed autistic people have a lower general belief in a just world compared to non-autistic people.
2. On average, people higher in autistic traits have a lower general belief in a just world than people lower in autistic traits.
3. The assumed negative relationship between formally diagnosed autism and the general belief in a just world is mediated by a lower personal belief in a just world, a higher external locus of control, and a lower proneness to self-deception.
4. The assumed negative relationship between autistic traits and the general belief in a just world is mediated by a lower personal belief in a just world, a higher external locus of control, and a lower proneness to self-deception.

For this aim, autistic and non-autistic people were recruited from online working platforms and asked to complete measures of autistic traits and the general belief in a just world. A subsample (including all formally diagnosed autistic people) also completed measures of the personal belief in a just world, locus of control, and self-deception. Furthermore, for additional analyses, age, gender, and intelligent reasoning ability were assessed as control variables.

## Method

### Participants

The diverse sample consisted of 588 US residents, recruited from online working platforms (Mechanical Turk, Prolific) to complete the measures of general belief in a just world, autistic traits, and reasoning ability. This included 60 individuals with a formal diagnosis of autism who were recruited via the pre-setting on Prolific. A subsample of 388 participants, including the 60 autistic participants, additionally completed measures of potential mediators (personal belief in a just world, locus of control, and self-deception). The participants' sociodemographic data are presented in Table 1. The participation was compensated by US$2 or US$5, depending on the number of measures (i.e., without or with the measures of mediator variables).

**Table 1:** Demographic information

	Total sample (*N* = 588)	Autistic participants^a^ (*n* = 60)	Non-autistic participants (*n* = 528)
Age (*M* ± 1*SD*)	37.68 ± 12.78	29.43 ± 9.20	38.62 ± 12.80
Gender			
Male	50.5%	50.0%	50.6%
Female	49.0%	48.3%	49.1%
Other	0.5%	1.7%	0.4%
Ethnicity			
Asian/Pacific Islander	5.6%	1.7%	6.1%
Black	8.5%	8.3%	8.5%
Hispanic or Latino	3.2%	3.3%	3.2%
Native American or American Indian	0.5%	0%	0.6%
White	77.4%	80.0%	77.1%
Mixed	3.6%	6.7%	3.2%
Other	1.2%	0%	1.3%
Highest level of education			
No high school diploma (or equivalent)	0.7%	1.7%	0.6%
High school diploma	32.8%	41.7%	31.8%
Bachelor’s degree	42.0%	31.7%	43.2%
Postgraduate degree	18.7%	13.3%	19.3%
Other	5.8%	11.7%	5.1%
Employment			
Self-employed	17%	8.3%	18.0%
State-employed	5.8%	1.7%	6.3%
Employed by private company or organization	52.7%	41.7%	54.0%
Homemaker	3.6%	1.7%	3.8%
Unemployed	14.3%	36.7%	11.7%
Other	6.6%	10.0%	6.3%

^a^Formally diagnosed

The sample size exceeded the minimum size of *N* = 548 necessary to detect at least a small-to-medium sized group difference (power analysis with G*Power 3.1, Faul et al. 2007; input: *t*-tests, difference between two independent group means, a priori, two-tailed, *d* = 0.40, α = 0.05, 1 − β = 0.80, allocation ratio *n*1/*n*2 = 9.00). The minimum effect size of 0.40 was chosen as the basis for calculation since Brysbaert (2019) concluded that it is the most reasonable estimate to use when looking for a non-negligible, useful, or theoretically meaningful effect if one has no further good evidence about the effect size. The sample allocation ratio of 9.00 was considered as the highest acceptable unevenness between the two categories of a dichotomous variable that does not provoke deflated statistical relationships with other variables (Tabachnick and Fidell 2007). Moreover, the sample size was more than twice the size that is required for stable estimates of correlations as high as the average published effect in social and personality psychology (*r* = .21; Schönbrodt and Perugini 2013). In addition, the subsample size of *n* = 388 participants was sufficient for mediation analyses in which both path coefficients of the indirect effect were at least small-to-medium-sized (*a* and *b* = 0.26; minimum *n* = 148), or even one of the two path coefficients was only small (a or b = 0.14; minimum *n* = 377 and 368 respectively; Fritz and MacKinnon 2007). Thus, the analyses were statistically well-powered.

Prior to the analyses, the data of 189 additionally recruited individuals were excluded due to potential double participation, incomplete data, or a failed attention check.

### Procedure and Measures

After giving informed consent, the participants provided sociodemographic information (e.g., age, gender) and indicated whether they were formally diagnosed with autism. This was followed by an attention check similarly applied in previous research (e.g., Bertrams and Schlegel 2020). Next, the subsequently described, established psychometric scales and the test of intelligent reasoning ability were presented in randomized order, each on a separate page. Finally, the participants were thanked, debriefed, and compensated.

#### Global Belief in a Just World Scale (GBJWS; Reich and Wang 2015)

This scale consists of seven items (e.g., “I feel that most people get what they are entitled to have”) answered on seven-point scales from 1 (*strongly disagree*) to 7 (*strongly agree*). For the total score, the item responses are summed, such that a higher total score represents a higher general belief in a just world. Cronbach’s α was 0.93 in the present study.

#### Autism Spectrum Quotient (AQ; Baron-Cohen et al. 2001)

The AQ has 50 items tapping different categories of autistic traits (e.g., “I often notice small sounds when others do not”) that are rated on a four-point scale from *definitely agree* to *definitely disagree*. Definitely/slightly agree and definitely/slightly disagree are awarded one and zero points, respectively (or zero/one points when items are reversed), which are summed to form a total score. Higher scores reflect higher autistic traits. In this study, Cronbach’s α was 0.85.

#### Personal Belief in a Just World Scale (PBJWS; Dalbert 1999)

The participants respond to seven items (e.g., “I believe that, by and large, I deserve what happens to me”) on seven-point scales from 1 (*strongly disagree*) to 7 (*strongly agree*). The total score is calculated by summing the answers to the items. A higher total score means a higher personal belief in a just world. For the sample in this study, Cronbach’s α was 0.92.

#### Internal–External Locus of Control Scale (I–E Scale; Rotter 1966)

The I–E Scale has 23 items (plus six filler items that are not analyzed). For each item, the participants choose between two response options (e.g., “Many of the unhappy things in people’s lives are partly due to bad luck” vs. “People’s misfortunes result from the mistakes they make”). While one of the response options stands for an internal locus of control (assigned zero points), the other option stands for an external locus of control (assigned one point). As the points are summed for a total score, higher values represent a higher external locus of control. In this study, Cronbach’s α was 0.76.

#### Self-deceptive Enhancement Subscale (SDE Subscale; Paulhus 1991)

This 20-item subscale is part of the Balanced Inventory of Desirable Responding, version 6. It measures the tendency for non-conscious self-deception according to how strongly the participants agree with exaggerated claims of positive cognitive attributes (e.g., “My first impressions of people usually turn out to be right”). The responses are given on a seven-point scale from 1 (*not true*) to 7 (*very true*). Points associated with each answer are summed across items; in accordance with Stöber et al. (2002), continuous scoring was applied in this study. Higher total scores indicate a higher proneness to self-deception. Cronbach’s α was 0.76 in the sample for this study.

#### Baddeley’s Grammatical Reasoning Test (BGRT; Baddeley 1968)

For this test of intelligent reasoning ability, the participants are presented with items consisting of a statement that describes the order of two letters (A and B) using the verbs “precede” or “follow” in the active or passive voice and positively or negatively (e.g., “A does not follow B,” “B is preceded by A”), followed by a letter pair (“BA” or “AB”). For each item, participants decide as quickly as possible whether the statement is true or false with respect to the letter pair. Participants have 3 min to solve as many items out of 64 as possible. The number of correctly solved items represents the level of reasoning ability. Cronbach’s α was 0.96 in this study.

### Analysis Strategy

In order to compare means, independent samples *t*-tests with two-tailed testing were applied. Pearson product-moment correlation coefficients with two-tailed testing were calculated to examine relationships. One exception to this was the test for differences in gender distribution between the autistic and non-autistic groups with the phi coefficient. In all analyses, a significance level α of 0.05 was used. To test the robustness of differences and relationships, bootstrapping was additionally applied. The mediation hypotheses were tested through confidence intervals based on bootstrapping, making use of the statistical tool PROCESS (Hayes 2013). Whenever bootstrapping was used in this study, it was bias-corrected and accelerated (BCa) and based on 1000 bootstrap samples.

In the preliminary analyses, potential differences between autistic and non-autistic people in age, gender distribution, and reasoning ability (as a proxy of intelligence) were examined. Relationships between autistic traits as well as general belief in a just world and these three control variables were also analyzed. In case any differences or relationships regarding the control variables emerged, additional analyses were conducted in which the respective variables were controlled for (analysis of covariance or multiple regression analysis). For statistical reasons, analyses involving gender were conducted without the three participants who declared to have a non-binary gender.

## Results

### Preliminary Analyses

#### Group Differences in Autistic Traits

As expected, formally diagnosed autistic people (*M* = 29.33, *SD* = 8.89) were higher in autistic traits than non-autistic people (*M* = 20.25, *SD* = 7.21; mean difference = 9.08, BCa 95% CI [6.84, 11.35], *t*[586] = 9.02, *p* < 0.001, *d* = 1.23).

#### Group Differences in Control Variables

As shown in Table 1, on average, the autistic group was younger than the non-autistic group (mean difference = − 9.18, BCa 95% CI [− 11.91, − 6.36], *t*[586] = 5.40, *p* < 0.001, *d* = 0.74). There was no difference in gender distribution between the autistic and the non-autistic group (Φ = − 0.001, *p* = 0.99, BCa 95% CI [− 0.09, 0.08]). Moreover, reasoning ability was not different between autistic (*M* = 32.77, *SD* = 14.61) and non-autistic people (*M* = 34.13, *SD* = 14.36; mean difference = − 1.36, BCa 95% CI [− 4.97, 2.84], *t*[586] = 0.70, *p* = 0.49, *d* = 0.10).

#### Relationships with Control Variables

The general belief in a just world did not correlate with age (*r*[586] = 0.07, BCa 95% CI [− 0.01, 0.16], *p* = 0.09). Women had a lower general belief in a just world (*r*[583] = − 0.13, BCa 95% CI [− 0.21, − 0.05], *p* = 0.002). A higher reasoning ability was associated with a lower general belief in a just world (*r*[586] = − 0.23, BCa 95% CI [− 0.31, − 0.16], *p* < 0.001).

Higher autistic traits correlated with lower age in the sample (*r*[586] = − 0.09, BCa 95% CI [− 0.17, − 0.01], *p* = 0.03). Autistic traits did not correlate with gender (*r*[583] = 0.001, BCa 95% CI [− 0.08, 0.09], *p* = 0.98). The higher the autistic traits, the higher was the reasoning ability (*r*[586] = 0.08, BCa 95% CI [0.002, 0.16], *p* = 0.04).

### Autistic Compared to Non-autistic People’s General Belief in a Just World (Hypothesis 1)

The formally diagnosed autistic individuals (*M* = 20.40, *SD* = 7.97) had a lower general belief in a just world than the non-autistic individuals (*M* = 27.37, *SD* = 9.70). The mean difference of − 6.97, BCa 95% CI [− 8.91, − 4.96], was significant (*t*[586] = 5.37, *p* < 0.001, *d* = 0.73).

#### Including Control Variables

Given that the autistic and the non-autistic groups did differ in age, and gender and reasoning ability were correlated with the general belief in a just world, the analysis was repeated with these variables as covariates. Again, autistic (*M*_adj_ = 20.85, *SE*_adj_ = 1.22) compared to non-autistic (*M*_adj_ = 27.36, *SE*_adj_ = 0.40) people’s general belief in a just world was significantly lower (mean difference = − 6.51, BCa 95% CI [− 8.68, − 4.32], *F*[1, 580] = 25.41, *p* < 0.001, η^2^_part_ = 0.04).

The covariate age was not significant (*p* = 0.13, η^2^_part_ = 0.004); women had a lower general belief in a just world than men (*p* = 0.001, η^2^_part_ = 0.02); and higher reasoning ability was related to a lower general belief in a just world (*p* < 0.001, η^2^_part_ = 0.06).

### Relationship Between Autistic Traits and General Belief in a Just World (Hypothesis 2)

As expected, higher autistic traits were associated with a lower general belief in a just world (*r*[586] = − 0.31, BCa 95% CI [− 0.38, − 0.24], *p* < 0.001). Both variables were also correlated in this way, separately, in the group of autistic people (*r*[58] = − 0.48, BCa 95% CI [− 0.67, − 0.26], *p* < 0.001) and the group of non-autistic people (*r*[526] = − 0.23, BCa 95% CI [− 0.31, − 0.15], *p* < 0.001).

#### Including Control Variables

Controlling for age, gender, and reasoning ability showed that autistic traits and the general belief in a just world were significantly negatively related beyond the control variables in the overall sample (*B* = − 0.34, BCa 95% CI [− 0.44, − 0.25], *SE B* = 0.05, β = − 0.28, *p* < 0.001), in the group of autistic people (*B* = − 0.37, BCa 95% CI [− 0.59, − 0.17], *SE B* = 0.10, β = − 0.42, *p* < 0.001), and in the group of non-autistic people (*B* = − 0.28, BCa 95% CI [− 0.39, − 0.18], *SE B* = 0.06, β = − 0.21, *p* < 0.001).

Age was positively related to the general belief in a just world in the overall sample (β = 0.08, *p* = .04), but not in the separated autistic and non-autistic groups (βs = 0.21 and 0.05, *p*s > 0.05). Gender and the general belief in a just world were related in the overall sample and in the non-autistic group (βs = − 0.13 and − 0.14, *p*s < 0.002 [with a lower general belief in a just world in women]), but not in the autistic group (β = − 0.06, *p* = 0.59). In all three regression analyses, a higher reasoning ability was related to a lower general belief in a just world (βs = − 0.21, − 0.29, and − 0.22, *p*s < 0.02). All overall regression models were significant (*p*s < 0.001).

### Mediation Analyses (Hypotheses 3 and 4)

#### Personal Belief in a Just World

Formally diagnosed autistic people (*M* = 27.53, *SD* = 8.45) had a lower personal belief in a just world than non-autistic people (*M* = 32.80, *SD* = 8.31; mean difference = − 5.27, BCa 95% CI [− 7.55, − 2.87], *t*[386] = 4.50, *p* < 0.001, *d* = 0.63). Moreover, the higher the autistic traits, the lower was the personal belief in a just world (*r*[386] = − 0.29, BCa 95% CI [− 0.39, − 0.19], *p* < 0.001). A lower personal belief in a just world was also associated with a lower general belief in a just world (*r*[386] = 0.69, BCa 95% CI [0.60, 0.76], *p* < 0.001).

Conforming the hypothesis, the personal belief in a just world significantly mediated the indirect effect (*ab*) of the autism group (formally diagnosed autistic vs. non-autistic) on the general belief in a just world, indicated by the fact that the respective bootstrap interval did not include the null (Hayes 2013; *ab* = − 3.80, *SE ab* = 0.88, BCa 95% CI [− 5.41, − 2.05]). Controlling for age, gender, and reasoning ability did not change the result (*ab* = − 3.16, *SE ab* = 0.88, BCa 95% CI [− 5.03, − 1.54]).

The indirect effect between autistic traits and the general belief in a just world was also mediated by the personal belief in a just world (*ab* = − 0.23, *SE ab* = 0.05, BCa 95% CI [− 0.32, − 0.14]). This result held after statistically controlling for age, gender, and reasoning ability (*ab* = − 0.21, *SE ab* = 0.04, BCa 95% CI [− 0.30, − 0.13]).

#### External Locus of Control

Autistic people (*M* = 15.28, *SD* = 4.79) had a higher external locus of control than non-autistic people (*M* = 12.46, *SD* = 4.11; mean difference = 2.82, BCa 95% CI [1.55, 4.09], *t*[386] = 4.77, *p* < 0.001, *d* = 0.67). Higher autistic traits were correlated with a higher external locus of control (*r*[386] = 0.29, BCa 95% CI [0.18, 0.39], *p* < 0.001). Furthermore, the higher the external locus of control, the lower was the general belief in a just world (*r*[386] = − 0.51, BCa 95% CI [− 0.58, − 0.43], *p* < 0.001).

External locus of control mediated the indirect effect between autism group and the general belief in a just world (*ab* = − 2.82, *SE ab* = 0.68, BCa 95% CI [− 4.24, − 1.60]). This mediation was also significant when age, gender, and reasoning ability were controlled for (*ab* = − 2.25, *SE ab* = 0.68, BCa 95% CI [− 3.62, − 0.90]).

Having an external locus of control also mediated the indirect effect between autistic traits and the general belief in a just world (*ab* = − 0.16, *SE ab* = 0.03, BCa 95% CI [− 0.23, − 0.10]). The mediation remained significant after statistically controlling for age, gender, and reasoning ability (*ab* = − 0.14, *SE ab* = 0.03, BCa 95% CI [− 0.21, − 0.08]).

#### Self-deception

Self-deception was lower in formally diagnosed autistic people (*M* = 77.12, *SD* = 11.82) than in non-autistic people (*M* = 83.79, *SD* = 14.12; mean difference = − 6.67, BCa 95% CI [− 9.83, − 3.07], *t*[386] = 3.44, *p* < 0.001, *d* = 0.48). In addition, higher autistic traits were associated with lower self-deception (*r*[386] = − 0.36, BCa 95% CI [− 0.44, − 0.28], *p* < 0.001). When self-deception was higher, the general belief in a just world was also higher (*r*[386] = 0.40, BCa 95% CI [0.29, 0.51], *p* < 0.001).

Self-deception was a significant mediator of the relationship between autism (formally diagnosed autistic people vs. non-autistic people) and the general belief in a just world (*ab* = − 1.60, *SE ab* = 0.50, BCa 95% CI [− 2.75, − 0.79]). This result remained robust after controlling for age, gender, and reasoning ability (*ab* = − 1.17, *SE ab* = 0.41, BCa 95% CI [− 2.07, − 0.42]).

There was also a significant mediation of the relationship between autistic traits and the general belief in a just world via self-deception (*ab* = − 0.15, *SE ab* = 0.03, BCa 95% CI [− 0.22, − 0.09]). The mediation was significant even after controlling for age, gender, and reasoning ability (*ab* = − 0.12, *SE ab* = 0.03, BCa 95% CI [− 0.18, − 0.07]).

## Discussion

### Study Findings

The results from this study revealed that, on average, formally diagnosed autistic people and people high in autistic traits possessed a lower general belief in a just world than people without an autism diagnosis or lower autistic traits. Thus, autistic individuals are less likely to believe in the view that people get what they deserve and deserve what they get. As expected, this relationship was mediated by a lower personal belief in a just world, a higher external (or lower internal) locus of control, and a lower tendency for self-deception. This pattern held even when controlling for the potential influences of age, gender, and intelligent reasoning ability.

### Implications

The findings have various implications. First, they deliver a novel mosaic piece regarding autistic personality. Whether one believes that the world is just or not is a fundamental basis from which perceptions and decisions are made in everyday life (Ross and Miller 2002). For instance, people with a lower belief in a just world are less socially accommodating (Lipkus and Bissonnette 1996) and less biased by others’ group memberships when judging them (Freeman 2006). A tendency toward less diplomacy and less biased information processing have also been found in autistic people or people high in autistic traits (Baron-Cohen et al. 2001; Brosnan et al. 2016; Karvelis et al. 2018; Lewton et al. 2019; Pellicano and Burr 2012). Such autistic characteristics could partly result from their lesser illusion of a just world. With a low general belief in a just world, one cannot rely on the forsaking of one’s self-interest to be rewarded in return, which lowers the perceived benefit of accommodation (Lipkus and Bissonnette 1996). Moreover, without the belief that people get what they deserve, group memberships (e.g., low socioeconomic status) are not considered valid deliverables of information about others’ traits (Freeman 2006). It is possible that some experiences and behaviors typical for autism and high autistic traits are not directly caused by the autistic traits but mediated by a relatively low general belief in a just world.

Furthermore, these results point to a crucial autistic strength, namely, the ability to see things in an unbiased, realistic manner. In line with this view, previous researchers have shown that autistic people are less susceptible to the framing effect (De Martino et al. 2008) and the conjunction fallacy (Morsanyi et al. 2010) and that they are even less likely to succumb to visual illusions induced by top-down processes such as the Shepard Illusion (Mitchell et al. 2010) and the Kanisza Triangle (Happé 1996). The general belief in a just world may be another kind of fallacy or illusion to which autistic people are relatively immune. The evidence from this study suggests that lower susceptibility to some biases is essentially based on a lower tendency for self-deception. This attribute, in combination with their tendency to be undiplomatic, may make autistic people and people high in autistic traits valuable consultants whenever important decisions are made. History shows that self-deception occurs even on a grand scale and can lead to catastrophic decisions if not cleared up (e.g., the Challenger disaster; Trivers 2000).

However, there is also a downside. The general belief in a just world is considered to be a positive illusion that can increase subjective well-being (Dalbert 1998; Yu et al. 2018). The same applies to self-deception in general (Hagedorn 1996). Therefore, the present findings point to a possible reason why people with relatively subtle behavioral manifestations of autism and people high in autistic traits tend to have lowered subjective well-being (Kapp 2018; Lord et al. 2020; Stimpson et al. 2020): They deceive themselves too little in a positive direction, that is, they are too realistic. This refers to a dilemma that was symbolically portrayed by the blue and red pills in ‘The Matrix.’ Taking the blue pill stands for shutting oneself off mentally from the troubling elements of reality, which involves ignorance; taking the red pill stands for an unembellished view of reality, which can result in considerable discomfort. In interventions designed to help autistic people, solutions should be sought that appreciate their less-biased view of themselves and the world while still recognizing this as one potential cause of distress. In addition, their lower personal belief in a just world and higher external (or lower internal) locus of control can be based on valid grounds (e.g., Maïano et al. 2016) and can also contribute to lowered subjective well-being (Dalbert 1999; Klonowicz 2001). Therefore, considering the manifestations of these variables and the underlying reasons should additionally be a part of mental health support services.

### Limitations and Future Research

The findings of this study are limited in generalizability, as they are based on a convenient online sample that is supposedly not representative of the various autistic people; particularly, autistic people with additional intellectual disabilities were very likely underrepresented. Moreover, for reasons of anonymity and data protection, the stated formal diagnoses of autism could not be verified online (e.g., via uploaded documents). These are important limitations that are common in autism research (e.g., Livingston et al. 2020; Ola and Gullon-Scott 2020). Another limiting methodological aspect of this study is that the present findings rest on cross-sectional data. Future research should overcome these drawbacks by applying more targeted methods of data collection and measures that are less dependent on verbal-report skills than the typical self-report questionnaires. Longitudinal designs may provide further insights into the assumed causal relationships (Duckworth et al. 2010).

It should also be mentioned that the conceptualization and measurement of autistic traits, which was used in this study, has recently been questioned (Mottron and Bzdok 2020). Possibly, the sum of several self-reported traits, considered as representing a specific point on a continuum of being more or less autistic, is actually not informative with regard to clinical autism. In other words, autistic traits (as measured by the AQ) and clinical autism could be on qualitatively different dimensions. It remains to be seen how the scientific debate develops in this respect and which concept of autistic traits prevails in the future. If necessary, parts of the results of the present study may have to be re-evaluated.

Furthermore, some assumptions that have been made still need empirical examination; for instance, future research should directly test whether the usual experiences of autistic people and people high in autistic traits actually cause a lower personal belief in a just world and a higher external locus of control. In addition, the consequences of a low general belief in a just world (e.g., lowered well-being, less victim-blaming) have been documented in the literature (Dalbert 1998; Hafer 2002; Lerner and Simmons 1966; van den Bos and Maas 2009; Yu et al. 2018); however, it is possible that the underlying psychological processes are different for autistic people. Therefore, further research is still needed to reveal the effects of a lower belief in a just world in autistic people and people high in autistic traits.

## Data Availability

The datasets generated for this study are available on request to the author.
